# Environmental impact of commonly used anaesthetic agents: systematic literature review with narrative synthesis

**DOI:** 10.1016/j.bjao.2024.100362

**Published:** 2024-12-24

**Authors:** Philippa Lloyd, Alexander J. Fowler, Anna Wozniak, William Rattenberry, Sara Scott, Vikas Tripurneni, Mark Earl, Rupert M. Pearse, Sarah-Louise Watson, Tom.E.F. Abbott, Sarah Hare

**Affiliations:** 1 Barts and The London School of Medicine and Dentistry, Queen Mary University of London, London, UK; 2 William Harvey Research Institute, Queen Mary University of London, London, UK; 3 Adult Critical Care Unit, The Royal London Hospital, Barts Health NHS Trust, London, UK; 4 Department of Anaesthesia, Nottingham University Hospitals, Nottingham, UK; 5 Department of Anaesthesia, Queen Elizabeth Hospital, Gateshead, UK; 6 Department of Anaesthesia, Barking, Havering and Redbridge University Hospitals NHS Trust, London, UK; 7 Department of Anaesthesia, University College London Hospitals NHS Foundation Trust, London, UK; 8 Department of Anaesthesia, The William Harvey Hospital, Ashford, UK

**Keywords:** anaesthetics, desflurane, environmental impact, inhaled, intravenous, life cycle analysis, propofol, sevoflurane

## Abstract

**Background:**

Increasing awareness of the potential environmental impact of volatile anaesthetic agents has stimulated increased use of total i.v. anaesthesia. However, consolidated comparative evidence of the environmental impact of anaesthetic agents across the whole life cycle is lacking.

**Methods:**

We performed a systematic review and narrative evidence synthesis of the environmental impact of anaesthetic agents stratified by drug life cycle. We searched MEDLINE (PubMed), Excerpta Medica dataBASE (EMBASE), Cumulative index to nursing and allied health literature (CINAHL), and DrugBank, from inception until 05 March 2023, for studies describing the environmental impact of anaesthetic drugs on the WHO essential medicine list. Independent review and data extraction were performed by pairs of reviewers. Data on any aspect of cradle-to-grave life cycle analysis were reported, with narrative synthesis grouped according to life cycle domains.

**Results:**

From 15 229 records, 42 unique reports met our inclusion criteria. The predominant environmental impact of inhalation anaesthetic agents is atmospheric release, contributing to global warming. This may be improved with the emergence of more efficacious scavenging and capture systems. Packaging and waste contributed most to the environmental impact of i.v. anaesthetic agents. There is increasing concern over the ecological impact of i.v. agents entering water sources, either by disposal of unused medication or through the excretion of drug post-administration.

**Conclusions:**

There is increasing concern about the global warming impact of inhalation anaesthetic agents. However, there are insufficient ‘cradle-to-grave’ comparative analyses of the environmental impact of i.v. and inhalation anaesthesia to form evidence-based conclusions. Further research is urgently needed to guide clinical practice.

Climate change represents a substantial threat to public health, with implications globally.^1^, ^2^, ^3^,^1^, ^2^, ^3^ Surface temperatures have increased by approximately 1°C since the Industrial Revolution, and the Intergovernmental Panel on Climate Change (IPCC) recommends that global greenhouse gas emissions must be substantially reduced by 2029 to limit global warming to 1.5°C above pre-industrial levels.^4^ Surface temperatures are projected to increase above the ‘safe threshold’ of 2°C this century, with conservative predictions estimating a 2–3°C increase by 2090, without policies and active change to meet the IPCC target.^2^^,^^4^

The combined annual release of carbon dioxide (CO_2_) from healthcare systems in USA, Australia, UK, and Canada's is at least 748 million metric tonnes.^5^ In 2015, the NHS contributed 4.6% of the greenhouse gas emissions of the entire UK.^6^ This is expected to worsen, with projected increases in NHS clinical activity in the coming decade.^5^^,^^7^ Each year more than 310 million operations are undertaken worldwide, with variable climate impact according to the type of anaesthesia and surgery.^8^^,^^9^ Volatile inhalation anaesthetic agents are halogenated fluorocarbons (HFCs) with high global warming potential (GWP) that are routinely released into the atmosphere as waste and are widely detectable in the atmosphere.^10^ In 2014, desflurane had the highest atmospheric concentration (0.30 parts per trillion), followed by sevoflurane (0.13 parts per trillion), isoflurane (0.097 parts per trillion), and halothane (0.0092 parts per trillion).^10^ The atmospheric lifetime of volatile anaesthetic agents varies; the longest is desflurane at 14 yr. This compares to centuries-millennia for carbon dioxide and 121 yr for nitrous oxide.^10^^,^^11^ Although scavenging of waste anaesthetic vapours is commonplace, to improve health and safety of operating theatre environments, the capture of waste products is not routine and these are released into the atmosphere.^12^ Volatile anaesthetic compounds are little changed by human metabolism and are excreted through the lungs. Therefore, the majority of administered volatile anaesthetic agents are released into the atmosphere.^10^ Although there are commercially available capture devices to reduce their release in the atmosphere, the technology is in its infancy with low efficacy. The main alternative to volatile anaesthesia is total i.v. anaesthesia, but its environmental impact is not fully understood.^13^, ^14^, ^15^, ^16^, ^17^,^13^, ^14^, ^15^, ^16^, ^17^

Increasing publicity and awareness of the potential environmental impact of anaesthetic volatile drugs has resulted in scrutiny of current anaesthetic practice and a re-evaluation of the most appropriate methods. Initiatives to promote green anaesthesia and reduce the climate impact of anaesthesia and surgery, such as a reduction in anaesthetic use or using i.v. anaesthetic agents instead, are increasing.^18^ However, evidence to support strategic and clinical decision making to minimise the climate impact of anaesthesia is lacking. To fully understand the environmental impact of anaesthetic medications, a full life cycle, or ‘cradle-to-grave’ analysis is required, including natural resource extraction, manufacturing, packaging, transportation, use, reuse, and waste management or recycling.^17^^,^^19^ We performed a systematic review and evidence synthesis to establish and consolidate the current knowledge and data available about the entire carbon footprint and life cycle analysis for anaesthetic drugs.

## Methods

We performed a systematic literature review and narrative synthesis. Our main objective was to describe the overall environmental impact of the whole life cycle of commonly used anaesthetic drugs. We selected drugs listed in the anaesthetic section of the World Health Organization (WHO) essential medicine list (Supplementary Table S1).^20^ Our protocol was developed before starting the review process. We report our findings in line with the preferred reporting items for systematic reviews and meta-analyses (PRISMA) reporting guidelines (see Supplementary material).^21^^,^^22^

### Search strategy

We developed our search strategy on an adapted PICO (population/patient/problem, intervention/exposure, comparison/control, outcome) format using a combination of terms referring to anaesthetic agents listed above and terms associated with environmental impact. We searched MEDLINE (PubMed), Excerpta Medica dataBASE (EMBASE), Cumulative Index to Nursing and Allied Health Literature (CINAHL) using the Health Database Advanced Search platform from inception until 05 March 2023. We also searched DrugBank, which is a database of chemical characteristics of many medications.^23^ The search terms used are listed in Supplementary Table S2. In addition, we searched the bibliographies of all review articles and included papers for additional relevant citations. Searches included articles in any language. We removed duplicates and managed records using Mendeley (New York, NY, USA).^24^

### Study selection

Initial record screening and full-text assessment were performed in duplicate by pairs of investigators (WR, SS, VT, ME, PL, SW). Studies were included if both investigators agreed that they met the prespecified inclusions. Discrepancies between reviewers were resolved by discussion or by senior author review (SH/TA). Studies were eligible for inclusion if they reported data on any aspect of the ‘cradle-to-grave’ life cycle of any anaesthetic medication listed in the WHO essential medicines list (desflurane, sevoflurane, halothane, isoflurane, nitrous oxide, propofol, ketamine, thiopentone, bupivacaine, lidocaine, atracurium, vecuronium, suxamethonium [succinylcholine], and neostigmine) and were written in English. The cradle-to-grave life cycle steps were: raw materials, manufacturing processes, packaging, distribution, disposal of packaging, disposal of medication after use, and excretion of medication and metabolites from the patient.^25^ Studies reporting direct contamination of the operating theatre environment were excluded, as were studies reporting no original data.

### Data extraction

We developed a data extraction tool before beginning the data extraction process. For each study we extracted: author, year of publication, medication studied, study methodology, data on manufacturing processes, packaging, distribution, medication disposal, packaging disposal, intraoperative medication excretion. For all medications, the effect of these medications on aquatic environment was screened for.

### Data synthesis

We grouped studies based on the medications that they investigated and the relevant life cycle domains that they explored. We describe the number of studies and their relevant findings. We did not undertake meta-analysis. The frequency with which each life cycle domain has been studied, for each medication, is presented.

### Quality assessment

All included articles were assessed for quality using the Mixed Methods Appraisal Tool (MMAT) Version 2018 (McGill University, Montreal, Canada).^26^ This assessment was independently performed in duplicate (PL and AW), results were collated, and discrepancies were resolved between the two reviewers. Two questions were used to screen studies, in accordance with the MMAT protocol, and continued to assessment if both of these criteria were fulfilled.^26^ Screening and assessment questions can be found in Table 1. Overall risk of bias was assessed as low risk if all questions had been answered ‘yes’, high risk if some questions had been answered ‘no’, and possible risk of bias if questions were answered ‘can't tell’ as well as ‘yes’.

**Table 1:**
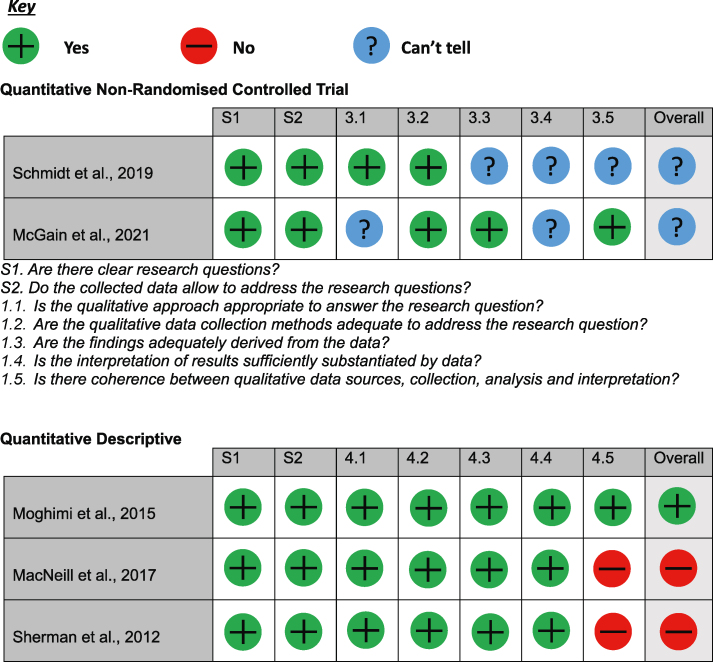

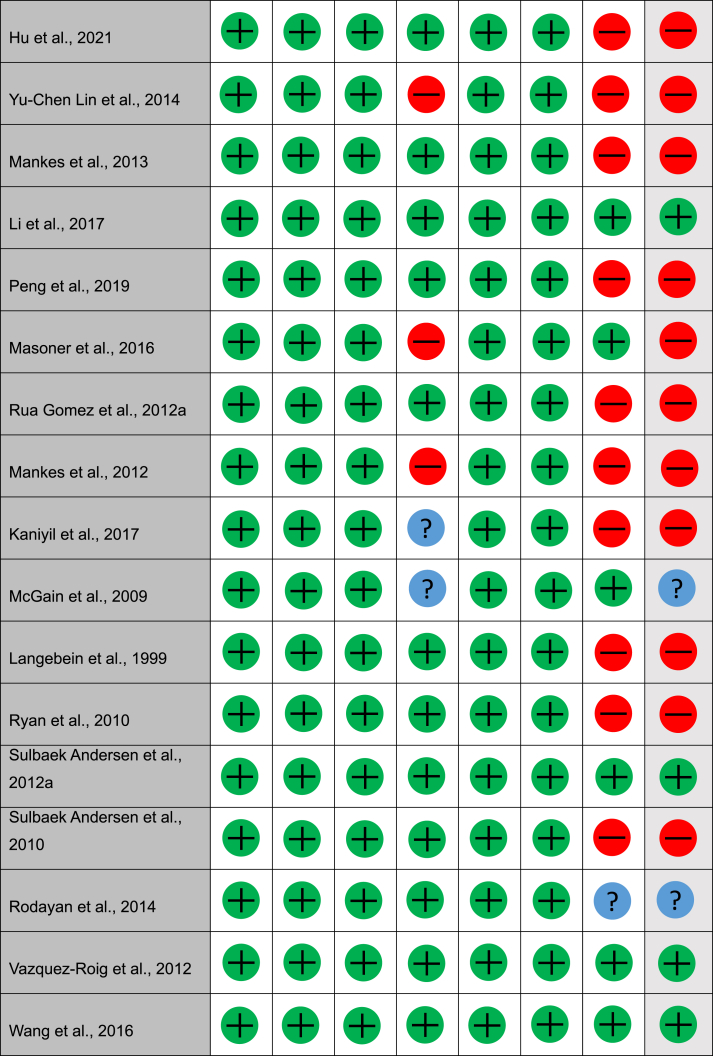

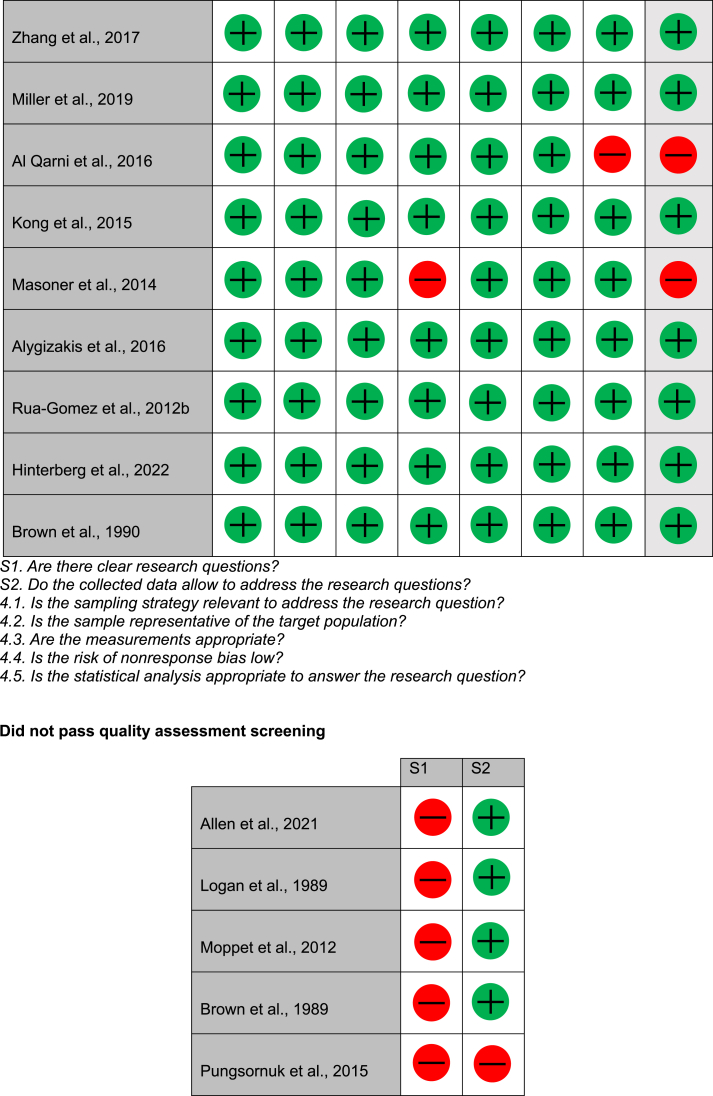

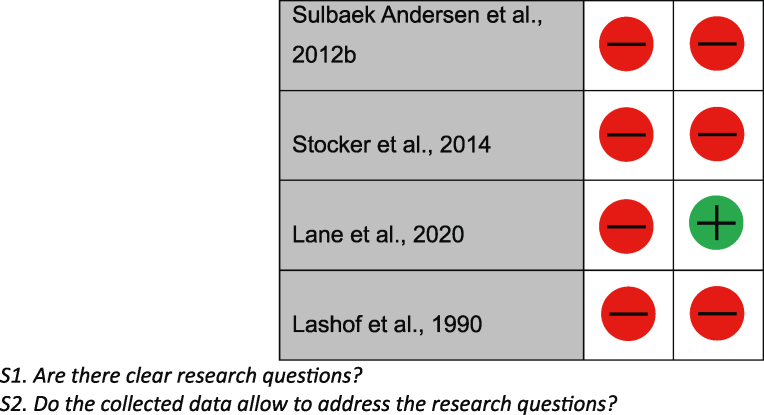
Results of the Mixed Methods Appraisal Tool (MMAT) quality assessment.

## Results

### Study selection

**Fig 1:**
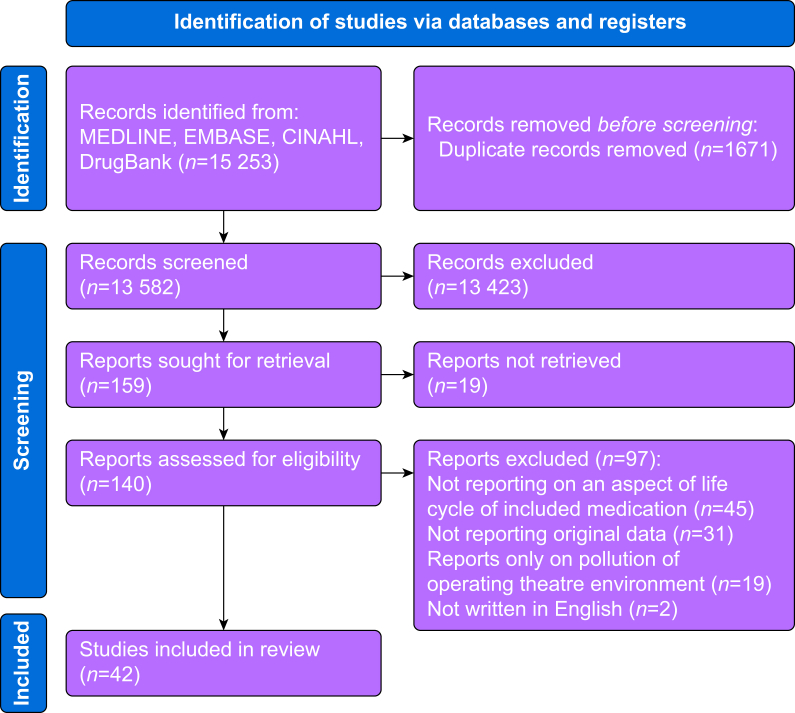
PRIMSA diagram illustrating the systematic review process.

We identified 15 229 records from the electronic search strategy, and a further 24 records through hand searches. After removal of duplicates, 13 582 records remained. The full texts of 140 studies were retrieved, of which 42 met the inclusion criteria. A summary of article selection is shown in Figure 1.

### Study characteristics

The characteristics of included studies are summarised in Table 2. Analysed manuscripts included environmental science papers (*n*=24), basic science papers (*n*=1), environmental impact assessments (*n*=7), review articles (*n*=3), and audits/quality improvement projects (*n*=2). Four articles were commentaries or letters to the editor, and one article was a poster abstract. Papers were heterogenous in methodology, results, and presentation.

**Table 2:**
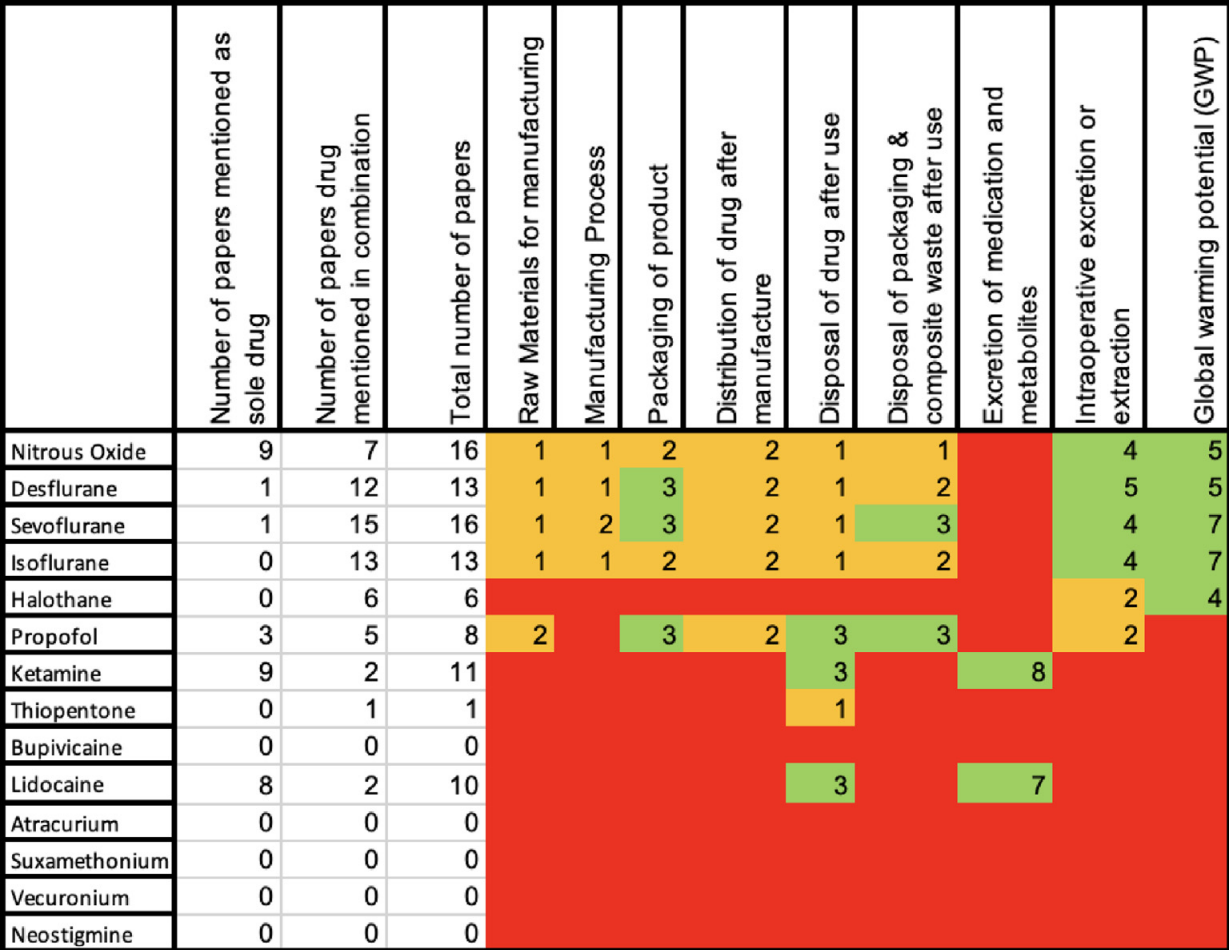
Characteristics and heatmap of included papers. Domains with no relevant papers are coded as red, with one to two papers are coded as yellow, with more than two papers are coded as green.

### Quality of evidence

During initial screening for the quality assessment, 9/42 did not have clear research questions or collect the correct data to address the research question and therefore were not analysed. Of the remaining studies, 2/42 were qualitative non-randomised controlled trials, 30/42 were qualitative descriptive studies, and 1/42 was not available. Of the two quantitative non-randomised controlled trials, both were answered either yes or ‘can't tell’ to the assessment questions; therefore, they were determined to have an overall possible risk of bias. Of the quantitative descriptive studies, 15/30 studies were found to have a high risk of bias, 2/30 had possible risk of bias, 13/30 had low risk of bias. These results can be found in Table 1.

### Life cycle domains

#### Raw materials for manufacturing

Two studies reported the raw materials used in the manufacture of medications. In 2021 Hu and colleagues^27^ demonstrated that differences in raw materials and method of synthesis impacts the carbon footprint of inhaled anaesthetic agents. Sevoflurane that is manufactured using tetrafluoroethylene has a five-fold increase in carbon footprint compared with sevoflurane that is manufactured using gas-phase catalytic fluorination of hexachloroacetone.^27^ Many pharmaceutical companies report sustainable sources for raw materials used to manufacture anaesthetic agents.^28^ In 2020 Lane^28^ examined the manufacture of propofol, which comprises 10% soybean oil. The authors reported that this is associated with deforestation of the Amazon basin.^28^ No studies compared the environmental impact of the raw materials used to manufacture i.v. *vs* volatile anaesthetic agents.

#### Manufacturing process

Two studies examined the manufacturing process of anaesthetic medications. One basic science paper proposed and tested a modified production method of sevoflurane with reduced reaction time and solvent requirement, which reduced the associated environmental impact.^29^ However, the extent to which pollution would be reduced if this modified process was implemented was not determined. In 2012 Sherman and colleagues^30^ modelled the production of desflurane, isoflurane, sevoflurane using data on catalysts and reagents derived from US patents. Their results showed that greenhouse gas emissions of sevoflurane production are roughly half of those for desflurane, while the emissions for isoflurane production is roughly half again.^30^ The manufacturing process of the medication was the largest contributor to the greenhouse gas emissions (not including release of waste gas) associated with the life cycle of desflurane and was the largest contributor for isoflurane and sevoflurane.^30^ However, this was a very small contribution compared with the environmental impact of the release of waste anaesthetic gases in this study, although no exact values were provided.^30^

#### Packaging of product

Three studies reported the environmental impact of packaging of medications from synthesis into distributable products. Hu and colleagues^27^ found that the amount of cardboard packaging used for propofol is nearly double that of sevoflurane, isoflurane, and desflurane (1.2968 kg and 0.67–0.7 kg, respectively, per kg of medication). A second study included the carbon footprint of packaging of isoflurane, desflurane, and sevoflurane in a life cycle analysis of greenhouse gas emissions, which showed that packaging contributed a very small proportion of overall emissions.^30^ In 2021 Allen and Baxter^31^ calculated that for 7 h of total i.v. anaesthesia using propofol, 0.443 kg of plastic and 0.472 kg of glass is required. This produces 1.44 kg and 0.42 kg CO_2_ equivalents, respectively, constituting 1.86 kg out of a total 3.2 kg (58.12%) for all aspects of use, including a life cycle assessment of the medication, packaging, waste incineration, and electricity usage.^31^ This suggests that packaging constitutes a large proportion of the environmental impact of total i.v. anaesthesia using propofol, but less for volatile agents.

#### Distribution of medication

Two studies examined the distribution of anaesthetic medications.^27^^,^^30^ Sherman and colleagues^30^ included road transport of anaesthetic medications in a modelling analysis, with anaesthetic gases being transported in lightweight polyvinyl chloride plastic containers and propofol transported in glass containers. Although they provided no specific values on the environmental impact of the supply chain, the analysis showed that transport makes up a very small proportion of the overall carbon footprint of anaesthetic medication use.^30^ A second study found that as most medications tend to be dispatched from a single logistic centre via road transport, the carbon footprint of transportation should be the same for most medications of the same weight and transport conditions.^27^ However, the authors did not consider differences in volume of medication required per patient, as accounted for by Sherman and colleagues.^27^ Nonetheless, this difference is likely negligible.

#### Disposal of medication after use

Six studies reported the environmental impact of medication disposal. One study observed that 28 950 mg of thiopentone, 23 330 mg of propofol, 11 700 mg of lidocaine, and 1110 mg of ketamine were wasted from the operating theatre complex of one US hospital over 5 weeks.^32^ In 2012 Mankes^33^ found that propofol accounted for 45% of the total medication wastage across eight US operating theatres making it the most widely wasted medication at the facility. This was greatly reduced (from 29.2 ml day^−1^ to 2.8 ml day^−1^ per bin) by eliminating the larger 50- and 100-ml bottles from the operating suites. One life cycle analysis examined wastage of propofol.^30^ The authors assumed a 50% propofol wastage based on previous literature.^30^ Although they state the total CO_2_ equivalent of the life cycles of each medication, they do not state the specific values for each domain, including disposal of medication.^30^ Nonetheless they do represent these values in a figure and disposal of medications is showed to represent a small proportion of the total CO_2_ equivalent of propofol.^30^ In addition, they state that unadministered propofol must be disposed of by incineration in accordance with pharmaceutical waste regulations and manufacturer recommendations, the environmental impact of which is unknown.^30^ Mankes and Silver^34^ in 2013 found that across two US healthcare facilities (2008–9), 22.2% (36.4 g) of ketamine was wasted. In 20124 Masoner and colleagues^35^ also found that lidocaine was present in 89% of 19 landfill sites sampled across the USA. A separate study examined leachate samples from 22 landfills and found that lidocaine was the most frequently detected chemical of emerging concern, present in 91% of samples at a maximum concentration of 47 900 ng L^−1^.^36^ Untreated leachate was continuously discharged from 10 landfill sites, six of which were unlined and discharged directly into groundwater, whereas one site discharged directly into a river.^36^

#### Disposal of packaging and consumables

Four studies reported the environmental impact of the disposal of packaging and composite waste. In 2009 McGain and colleagues^37^ reported that anaesthesia waste accounted for 25% of the total operating theatre waste at one large Australian hospital. More than half of this waste was recyclable but was not recycled owing to infection control reasons.^37^ An environmental impact assessment by MacNeill and colleagues^38^ in 2017 found that three hospitals in Canada, USA, and UK each produced an average of 267 829 kg of waste per year (606 423 CO_2_ equivalent) owing to surgical consumables. However, this was not specific for anaesthetic packaging waste, rather all surgical consumables and packaging.^38^ A life cycle assessment by McGain and colleagues^39^ in 2021 reported that the greatest component of CO_2_ equivalent emissions from general anaesthesia and spinal anaesthesia is sevoflurane (35%), followed by single-use plastics (20–25%), electricity (15%), and pharmaceuticals (8%). Conversely, in 2012 Sherman and colleagues^30^ concluded that packaging disposal constitutes a minimal proportion of the total greenhouse gas emissions of anaesthetic medications' life cycles after accounting for the carbon footprint of waste management.

#### Excretion of medication and metabolites

Sixteen studies reported the environmental impact of excreted anaesthetic medications and their metabolites. In 2014 Lin and colleagues^40^ analysed 36 water sources in Taiwan, 13 of which were effluents from hospitals, and found ketamine and norketamine to be present at similar concentrations to that found in the urine of patients administered the medication. Two studies in Beijing observed ketamine and its metabolites in sewage effluents and surface waters.^41^^,^^42^ Other studies found ketamine in 36 rivers in North China and in irrigation channels in Spain.^43^^,^^44^ Ketamine concentrations were highest in the centres of urban areas. A study by Peng and colleagues^45^ in 2019 detected 0.14–1.12 ng L^−1^ of ketamine in drinking water samples from East Anglia, UK. In 2017 Li and colleagues^46^ found that at environmentally relevant concentrations, ketamine can have detrimental effects on the reproduction of *Daphnia magna*, a species of water flea. At higher concentrations, ketamine causes acute biological toxicity to a range of aquatic organisms.^46^ Ozonisation, which may be used to degrade ketamine in water sources, has been shown to have a 16% removal rate from fresh water and 5% removal rate from wastewater.^47^ Several studies found lidocaine at environmentally relevant concentrations in wastewater from households and hospitals, at water treatment plants and in rivers/canals in Germany and China, and also in surface water and offshore sea water in Greece.^48^, ^49^, ^50^, ^51^,^48^, ^49^, ^50^, ^51^ Lidocaine was found in high concentrations within aquatic invertebrates in the UK.^52^ Three studies showed that these medications are only partially removed at wastewater treatment plants, with maximum efficacies of 50–64% for lidocaine and low (often negative) rate for ketamine, and a large variation between plants.^41^^,^^48^^,^^53^ Lidocaine has been shown to have a 31-h half-life in surface waters, thus efficient removal is necessary.^54^ There is limited research into the excretion of propofol and its metabolites, despite being the most commonly used i.v. medications. Propofol is very toxic to the aquatic environment, with a persistence, bioaccumulation, and toxicity (PBT) index hazard score of 6 out of a possible 9.^33^ In 2015 Campbell and Pierce^55^ concluded that owing to its metabolism, very little of an administered propofol dose is excreted and may enter the aquatic environment, which may highlight why it is an area of little concern. However, this does not account for the impact of unadministered propofol, which must be incinerated.^30^

#### Intraoperative excretion of waste gases

Six studies reported the environmental impact of intraoperative excretion of waste anaesthetic gases. In 2012 Moppett^56^ reported that only 25% of halothane, 3% of sevoflurane, 0.2% of isoflurane, and 0.02% of desflurane is metabolised by the cytochrome p450 enzymes, thus a large proportion is excreted. Sherman and colleagues^30^ reported that waste anaesthetic gas release accounted for the majority of the total greenhouse gas emissions associated with the life cycle of desflurane, isoflurane, and sevoflurane. Logan and Farmer^57^ estimated that in the UK, healthcare services released 1×10^9^ L of nitrous oxide per year. In 2021 Hu and colleagues^27^ examined the effectiveness of vapour capture technology and found that general anaesthesia, maintained in an oxygen/air mix at 0.5 L min^−1^ gas flow rate, using a system with a 70% gas recycling rate reduces the carbon footprint of sevoflurane to be similar to that of propofol. However, another study examined the efficiency of inhaled anaesthetic gas recapture systems and found that only 25% of administered desflurane was recaptured.^58^ They hypothesised that a significant residual amount of desflurane excreted after extubation was not captured.^58^ In 2019 Schmidt and colleagues^59^ validated the safety and feasibility of a membrane-based product (memsorb™; DMF Medical Inc., Halifax, NS, Canada) as an alternative to chemical absorbers under low-flow, minimal-flow, and metabolic-flow conditions for use in scavenging systems. As this technology continuously and passively functions over 10–12 months compared with 1 day for the chemical absorbers, this could reduce environmental impact of the technology as a result of a reduction in absorber waste and absorber transportation.^59^

Volatile anaesthetic agents are routinely released from operating theatres to mitigate occupational exposure, which is a legal requirement in many jurisdictions. The high GWPs, long atmospheric lifetimes, and low rate of metabolism of these compounds result in a quantifiable environmental impact.

## Discussion

We have undertaken a systematic review of life cycle analyses of anaesthetic medications with a narrative evidence synthesis. Although anaesthesia clearly has an environmental impact, we found limited evidence of comprehensive life cycle analyses comparing types of general anaesthesia. Therefore, it is difficult to conclude whether one mode of anaesthesia has a lower environmental impact overall compared with another. The most concerning finding is the impact of waste anaesthetic agents, as the majority of administered volatile anaesthetic agents are released into the atmosphere, because of the low rate of metabolism and the low prevalence of capture systems. The greenhouse effect of these compounds exists, highlighting an important area for improvement. I.V. anaesthetic agents have been found globally at environmentally relevant concentrations in wastewater and efficacy of removal systems has been reportedly low. Propofol has been found to be a widely wasted medication, with large volumes entering landfill sites and leaching into local water sources. Additionally, there are little publicly available life cycle analyses regarding the manufacturing processes of propofol. It is possible that the production plants consume large amounts of water, fuel, and raw materials, and produce large amounts of waste. Waste resulting from anaesthetic packaging and consumables has shown to contribute considerably to the total waste of healthcare facilities and the resulting environmental impact of this is poorly reported. Distribution of anaesthetic agents seems to contribute similarly between the two classes. Packaging appears to contribute a much greater proportion of the environmental impact of i.v. anaesthetic agents than that of volatile anaesthetic agents. This is because of the higher overall environmental impact of inhaled anaesthetic agent, and because greater duration of anaesthesia can be delivered using the volatile anaesthetic per unit volume than propofol in glass vial packaging. However, the contribution of packaging to the total environmental impact of these medications is unknown. Without evidence comparing the impact of anaesthetic agents across the whole medication life cycle, strategic decision-making to improve the environmental impact of anaesthesia and surgery may be impaired. More research is required to support evidence-based decisions to lessen the environmental impact of anaesthetic practice.

Although GWP_100_ is widely used to compare the global warming impact of various gases, there is recent debate as to whether this method may exaggerate the radiative effect of these compounds.^60^ Slingo and Slingo^60^ argue that radiative forcing may be a better comparative unit, which avoids the confounding of atmospheric lifetime with GWP, and depends only on the present atmospheric concentration of the gas. This may be a more complex measure than GWP and better illustrate the true global and regional climate change occurring as a result of each gas, accounting for non-uniform spatial distribution.^60^ Radiative forcing of volatile anaesthetic gases is only 0.01–0.02% of that of CO_2_, which would support a greater focus on efforts to mitigate carbon emissions of surgery and healthcare rather than reducing the use of volatile anaesthetics, so as not to adversely affect patient outcomes.^60^ However, Sherman and colleagues^61^ in 2021 rebut the arguments made by Slingo and Slingo^60^ in the same year, stating that GWP as a measure in fact does take into account atmospheric residence time, thus their critique may be flawed. Further, the established effects of halogenated compounds on the atmosphere may be mitigated by simple measures and adjustments, the responsibility for which lies with anaesthetists ‘as leaders’.

This study has both strengths and limitations. Although the methodology of this review is less robust than a quantitative meta-analysis, the heterogeneity and small quantity of data in each area was too limited to support this approach. A robust methodology with a broad range of search terms was used, so it is likely that the majority of relevant papers were included; however, it is possible that some research from non-English language sources or from grey literature were missed. Screening was performed by pairs of reviewers, which should minimise selection bias. We did not identify any studies that reported a full cradle-to-grave life cycle and environmental impact of any individual medication. Nor did we identify any analyses that compared all commonly used anaesthetic medications in any individual domain of environmental impact such as global warming. Therefore, it is not possible to make any robust comparison between the common alternative anaesthetic choices (such as inhalation anaesthesia *vs* i.v. anaesthesia *vs* regional anaesthesia) because, at this time, the evidence is not available to assess the environment impact of any counter-factual options. Similarly, there were multiple included medications which had no studies reporting on any aspect of their life cycles, as shown in Table 2. Therefore, there may be potential for great improvement in the environmental impact of the life cycles of these medications. However, the literature does not yet exist to explore this. Life cycle analysis has previously been explored for anaesthesia and surgery in a limited fashion. In 2021 Drew and colleagues^62^ performed a systematic review examining a comparable number of papers to this study (44), but with a wider focus, not limited to pharmaceuticals studied in this analysis, which provides a more in-depth review of the topic. Similar to the article by Drew and colleagues, McGain and colleagues^17^ and Roscioli and colleagues^63^ provide varied reviews that considered the climate impact of anaesthesia drugs as a component of perioperative surgical care, but without a specific focus on pharmaceuticals or without a life cycle assessment approach. Although there are several environmental science analyses of the GWP of nitrous oxide, desflurane, sevoflurane, and isoflurane, there is variation between techniques of analysis (e.g. ‘cloudy’ *vs* ‘non-cloudy’ calculations) and assumptions of volumes of gas and subsequent impact, making it difficult to directly compare findings. Our analysis may not have included studies, which observed a life cycle aspect of anaesthetics in general but did not report on any anaesthetic agents specifically. However, it is likely that there will have been very few studies to which this would apply. In addition, there are many aspects of the wider patient journey that are outside of the scope of the ‘cradle-to-grave’ analysis model, such as the ability to conduct preoperative clinics via telemedicine or the impact of travel for patients who experience same-day cancellations. These factors will exert their own environmental impacts and may vary greatly between modalities. It may be necessary to consider any such aspects within the overall environmental impact analysis of an anaesthetic medication to provide a complete picture for comparison.

This review highlights the lack of evidence surrounding the environmental impact of the full life cycles of anaesthetic medications. Although the release of waste anaesthetic gases and the wastage of medication and packaging of i.v. medications appear to contribute most greatly to these medications' overall impact, there are no cradle-to-grave life cycle analyses confirming or quantifying this. Similarly, although published research suggests that volatile anaesthetic agents exert a greater environmental impact than i.v. agents, the small number of existing life cycle analyses for anaesthetic medications suggests that any policy changes because of environmental concerns would be based on incomplete or limited data, and therefore may produce unintended harmful consequences. Therefore, we call for further and more in-depth comparative research resulting in comprehensive life cycle analysis data, including GWP, CO_2_ equivalents for each stage of the life cycle, and other factors such as landfill waste and water contamination. This would allow strategic changes to anaesthetic practice to be made, with the full extent of the impact on the environment of these decisions being understood.

## Authors' contributions

Study design: SH, AJF

Data collection: PL, AJF, WR, SS, VT, SLW

Interpretation: PL, TA, SH

Writing the first draft of the manuscript: PL, AJF, TA, SH

Quality assessment: PL, AW

Revision for important intellectual content and approved the final version: all authors

Access to the data and act as guarantors: PL, SH

## Declaration of interests

TA is supported by an NIHR clinical lectureship; has received research funding from Barts Charity, the Academy of Medical Sciences, The Royal College of Anaesthetists, and *British Journal of Anaesthesia*; has received honoraria from MSD and Edwards Life Sciences; and is Social Media Editor of the *British Journal of Anaesthesia*. AJF has received funding from NIHR DRF 2018-11-ST2-062. RMP has received honoraria, research grants, or both from Edwards Lifesciences, Intersurgical, and GlaxoSmithKline within the past 5 yr and holds editorial roles with the *British Journal of Anaesthesia* and the *British Journal of Surgery*. PL, WR, SS, VT, SLW, and SH have no conflicts of interest to declare.
