# Supplementary material for: Environmental impact of commonly used anaesthetic agents: systematic literature review with narrative synthesis
Source: BJA Open. 2024 Dec 24;13:100362. doi: 10.1016/j.bjao.2024.100362 (PMC11732243; doi:10.1016/j.bjao.2024.100362)
Supplement: Multimedia component 1 [file mmc1.docx]

**Environmental impact of commonly used anaesthetic agents: Systematic literature review with narrative synthesis**

Philippa Lloyd^1^, Alexander J. Fowler^2^, Anna Wozniak^3^, William Rattenberry^4^, Sara Scott^5^, Vikas Tripurneni*^6^*, Mark Earl^7^, Rupert. M. Pearse^2^, Sarah-Louise Watson^1^, Tom. E. F Abbott^2§^, Sarah Hare^§8^

§ joint senior authors

**SUPPLEMENTARY FILE**


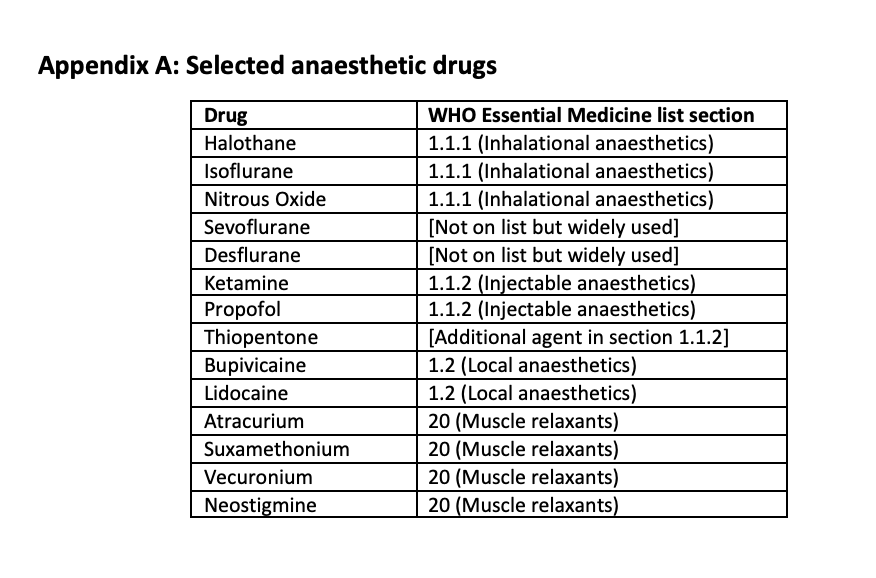
**Supplementary Table 1** - World Health Organisation List of Essential Medicines, 21^st^ List, 2019^20^

**Supplementary Table 2** – search terms


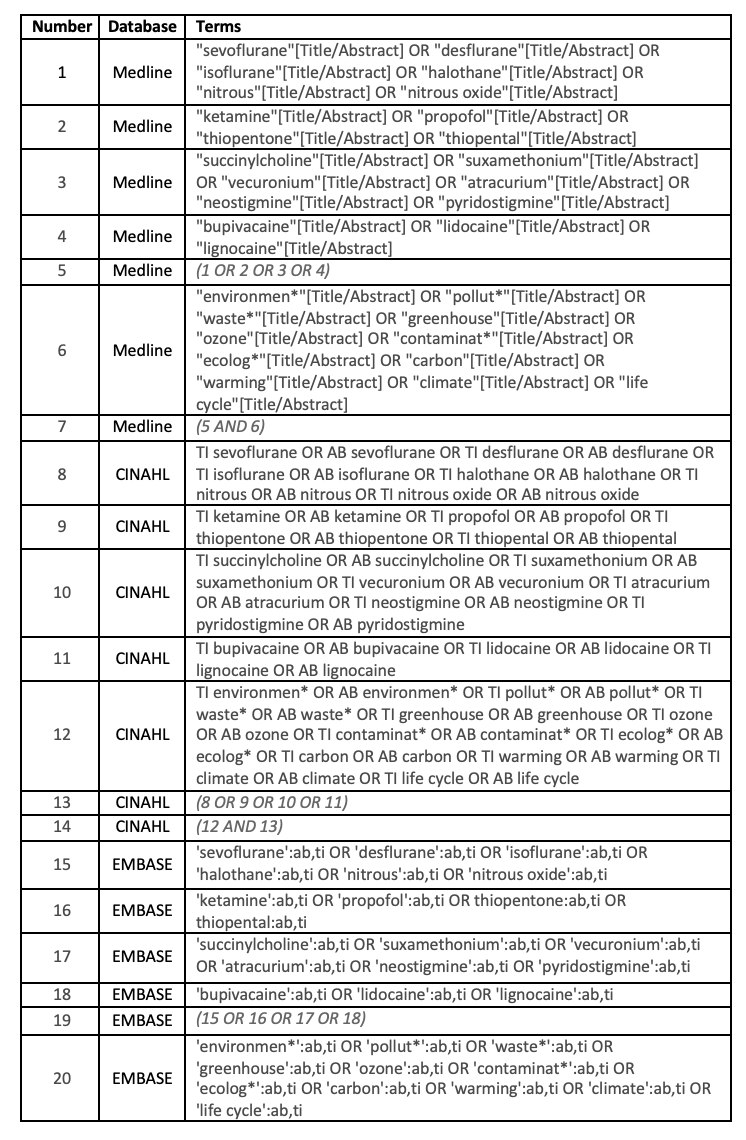


| **Findings as reported in this review** | Three studies showed these medications are only partially removed at wastewater treatment plants, with maximum efficacies of 50-64% for lidocaine and low (often negative) rate for ketamine, as well as a large variation between plants^5-7^. | Allen et al. (2021) calculated that for seven hours of total intravenous anaesthesia using propofol, 0.443 kg of plastic and 0.472 kg of glass is required^8^. This produces 1.44 kg and 0.42kg CO_2_ equivalents respectively, constituting 1.86 kg out of a total 3.2 kg (58.12%) for all aspects of use, including a life cycle assessment of the medication, packaging, waste incineration and electricity usage^8^. | Several studies found lidocaine at environmentally relevant concentrations in wastewater from households and hospitals, at water treatment plants and in rivers/canals in Germany and China, as well as in surface water and offshore sea water in Greece^5,9-11^ | However, another study examined the efficiency of inhaled anaesthetic gas recapture systems and found that only 25% of administered desflurane was recaptured^12^ | Hu et al. (2021) demonstrated that differences in raw materials and method of synthesis impacts the carbon footprint of inhaled anaesthetic agents^13^. Sevoflurane manufactured using tetrafluoroethylene has a five-fold increase in carbon footprint compared to sevoflurane manufactured using gas-phase catalytic fluorination of hexachloroacetone^13^.  Hu et al. (2021) found that the amount of cardboard packaging used for propofol is nearly double that of sevoflurane, isoflurane and desflurane (1.2968 Kg and 0.67-0.7 Kg respectively per Kg of medication)^13^  A second study found that since most medications tend to be dispatched from a single logistic centre via road transport, the carbon footprint of transportation should be the same for most medications of the same weight and transport conditions^13^. However, the authors did not consider differences in volume of medication required per patient, as accounted for by Sherman et al. (2012)^13^.  Hu et al. (2021) examined the effectiveness of vapour capture technology and found that general anaesthesia, maintained in an oxygen/air mix at 0.5L/min gas flow rate, using a system with a 70% gas recycling rate reduces the carbon footprint of sevoflurane to be similar to that of propofol^13^ | One study observed that 28,950 mg of thiopentone, 23,330 mg of propofol, 11,700 mg of lidocaine and 1,110 mg of ketamine were wasted from the operating theatre complex of one US hospital over 5 weeks^14^ | Several studies found lidocaine at environmentally relevant concentrations in wastewater from households and hospitals, at water treatment plants and in rivers/canals in Germany and China, as well as in surface water and offshore sea water in Greece^5,9-11^ | Many pharmaceutical companies report sustainable sources for raw materials used to manufacture anaesthetic agents^15^. Lane et al (2020) examined the manufacture of propofol, which comprises 10% soybean oil^15^. The authors reported that this is associated with deforestation of the Amazon basin^15^. |
| --- | --- | --- | --- | --- | --- | --- | --- | --- |
| **Relevant findings** | Average removal rate of lidocaine from 2 wastewater removal plants in Saudi Arabia was 64%, but with large variation (28% and >99%) | 7 hours of propofol TIVA produces 3.2kg CO2 equivalent, with 1.44kg and 0.42 kg from plastic and glass production respectively. This is compared to 820.2kg and 69.9kg total CO2 emissions for desflurane and sevoflurane respectively | Lidocaine was detected at concentrations of < 0.01–12.8 ng L^− 1^ in seawater of the Eastern Mediterranean Sea | 25% of administered desflurane is recaptured by recapture systems | Desflurane has the highest carbon footprint, due to the release do waste gases. Raw materials and synthesis method used hold an impact on the carbon footprint of inhaled agents. Packaging constitutes a higher proportion of the emissions of intravenous anaesthetic agents than inhaled. Distribution should produce similar environmental impact between the two anaesthetic modalities. With a recapture system with a 70% recapture rate, sevoflurane has a similar carbon footprint to propofol | 28,950 mg of thiopentone, 23,330 mg of propofol, 11,700 mg of lidocaine and 1,110 mg of ketamine were wasted from the operating theatre complex of one US hospital over 5 weeks | Across 20 surface water samples from Tianjin, North China, lidocaine was observed across 100% of samples, with an average concentration of 98 ng L^−1^ | Propofol comprises 10% soybean oil. Although manufacturers claim that the sources are sustainable, no sources were disclosed |
| **Life Cycle Domain(s) Studied** | Excretion of medication and metabolites | Packaging of product | Excretion of medication and metabolites | Intraoperative excretion or extraction | Packaging of product, Distribution of drug, Intraoperative excretion or extraction | Disposal of drug | Excretion of medication and metabolites | Raw materials |
| **Studied Drug(s)** | Lignocaine | Desflurane, Sevoflurane, Propofol | Lignocaine | Desflurane | Nitrous Oxide, Desflurane, Sevoflurane, Isoflurane, Propofol | Ketamine, Propofol, Thiopentone, Lignocaine | Lignocaine | Propofol |
| **Type of study** | Environmental Science paper | Correspondence | Environmental Science paper | Correspondence | Environmental Impact Assessment | Audit | Environmental Science paper | Correspondence |
| **Authors** | Al Qarni et al., 2016 | Allen et al., 2021 | Alygizakis et al., 2016 | Hinterberg et al., 2022 | Hu et al., 2021 | Kaniyil et al., 2017 | Kong et al., 2014 | Lane et al., 2020 |

**Supplementary Table 3** – Characteristics of included papers and key relevant findings

| **Findings as reported in this review** | Li et al. (2017) found that at environmentally relevant concentrations, ketamine can have detrimental effects on the reproduction of Daphnia magna, a species of water flea^16^. At higher concentrations ketamine causes acute biological toxicity to a range of aquatic organisms^16^ | Lin et al. (2014) analysed 36 water sources in Taiwan, 13 of which were effluents from hospitals and found ketamine and norketamine to be present at similar concentrations to that found in the urine of patients administered the medication^17^ | Logan et al. estimated in the UK, healthcare services released 1x10^9^ L of nitrous oxide per year^18^ | An environmental impact assessment by MacNeill et al. (2017) found that three hospitals in Canada, USA and UK each produced an average of 267,829 kg of waste per year (606,423 CO_2_ equivalent) due to surgical consumables^19^. However, this was not specific for anaesthetic packaging waste, rather all surgical consumables and packaging^19^ | Mankes et al. (2012) found that propofol accounted for 45% of the total medication wastage across eight US operating theatres making it the most widely wasted medication at the facility^20^. This was greatly reduced (from 29.2 mL/day/bin to 2.8 mL/day/bin) by eliminating the larger 50 and 100 mL bottles from the operating suites^20^ | Mankes et al. (2013) found that across two US healthcare facilities (2008-2009), 22.2% (36.4 g) of ketamine was wasted^21^. | Masoner et al. (2014) also found that lidocaine was present in 89% of 19 landfill sites samples across the US^22^ | A separate study examined leachate samples from 22 landfills and found that lidocaine was the most frequently detected chemical of emerging concern, present in 91% of samples at a maximum concentration of 47,900 ng/L^23^. Untreated leachate was continuously discharged from ten landfill sites, six of which were unlined and discharged directly into groundwater, while one site discharged directly into a river^23^. | McGain et al. (2009) reported that anaesthesia waste accounted for 25% of the total operating theatre waste at one large Australian hospital ^24^. Over half of this waste was recyclable but was not recycled due to infection control reasons^24^ |
| --- | --- | --- | --- | --- | --- | --- | --- | --- | --- |
| **Summary of findings** | At environmentally relevant concentrations, ketamine has detrimental effects on the reproduction of Daphnia magna (water flea) and at higher concentrations it can cause acute biological toxicity to a range of aquatic organisms | 36 water sources in Taiwan found ketamine and norketamine to be present at similar concentrations to that found in the urine of patients administered the drug. Maximum ketamine concentration is 10 μg/L | UK healthcare services release 1x10^9^ L of nitrous oxide per year | Three hospitals in Canada, USA and UK each produced an average of 267,829 kg of waste per year (606,423 CO_2_ equivalent) due to surgical consumables | Across 8 US operating theatres, propofol accounted for 45% of the total drug wastage and was the most widely wasted medication at the facility | Across two US healthcare facilities, 22.2% (36.4 g) of ketamine was wasted between 2008 and 2009 | Lidocaine was present in 89% of landfill sites in the US | Across 22 US landfills, lidocaine was the most frequently detected chemical of emerging concern, present in 91% of samples at a maximum concentration of 47,900 ngL^-1^ | Anaesthetics waste accounted for 25% of the total operating theatre waste at one large Australian hospital |
| **Life Cycle Domain(s) Studied** | Excretion of medication and metabolites | Excretion of medication and metabolites | Intraoperative excretion or extraction | Disposal of packaging | Disposal of drug | Disposal of drug | Disposal of drug | Disposal of drug | Disposal of packaging |
| **Studied Drug(s)** | Ketamine | Ketamine | Halothane, enflurane, isoflurane, Nitrous Oxide | Desflurane, Sevoflurane, Isoflurane | Propofol | Ketamine | Lignocaine | Lignocaine | Propofol |
| **Type of study** | Environmental Science paper | Environmental Science paper | Review article | Environmental Impact Assessment | Environmental Science paper | Environmental Science paper | Environmental Science paper | Environmental Science paper | Audit |
| **Authors** | Li et al., 2017 | Lin et al., 2014 | Logan et al., 1989 | MacNeill et al., 2017 | Mankes et al., 2012 | Mankes et al., 2013 | Masoner et al., 2014 | Masoner et al., 2016 | Mcgain et al., 2009 |

| **Findings as reported in this review** | A life cycle assessment by McGain et al. (2021) reported that the greatest component of CO_2_ equivalent emissions from general anaesthesia and spinal anaesthesia is sevoflurane (35%), followed by single-use plastics (20-25%), electricity (15%) and pharmaceuticals (8%)^25^. | Lidocaine was found in high concentrations within aquatic invertebrates in the UK^26^ | One basic science paper proposed and tested a modified production method of sevoflurane with reduced reaction time and solvent requirement, which reduced the associated environmental impact^27^. However, the extent to which pollution would be reduced if this modified process was implemented was not determined. | Moppett et al (2012) reported that only 25% of halothane, 3% of sevoflurane, 0.2% of isoflurane and 0.02% of desflurane is metabolised by the cytochrome p450 enzymes, thus a large proportion is excreted^28^ | A study by Peng et al. (2019) detected 0.14–1.12ng/L ketamine in drinking water samples from East Anglia, UK^29^ | Ozonisation, which may be used to degrade ketamine in water sources has been shown to have a 16% removal rate from fresh water and 5% removal rate from wastewater^30^. | Several studies found lidocaine at environmentally relevant concentrations in wastewater from households and hospitals, at water treatment plants and in rivers/canals in Germany and China, as well as in surface water and offshore sea water in Greece^5,9-11^. Lidocaine was found in high concentrations within aquatic invertebrates in the UK^26^. Three studies showed these medications are only partially removed at wastewater treatment plants, with maximum efficacies of 50-64% for lidocaine and low (often negative) rate for ketamine, as well as a large variation between plants^5-7^. | Several studies found lidocaine at environmentally relevant concentrations in wastewater from households and hospitals, at water treatment plants and in rivers/canals in Germany and China, as well as in surface water and offshore sea water in Greece^5,9-11^. | Schmidt et al. (2019) validated the safety and feasibility of a membrane-based product (memsorb™, DMF Medical Inc., Halifax, NS, Canada) as an alternative to chemical absorbers under low-flow, minimal-flow and metabolic-flow conditions for use in scavenging systems^31^. |
| --- | --- | --- | --- | --- | --- | --- | --- | --- | --- |
| **Relevant findings** | the greatest component of CO_2_ equivalent emissions from general anaesthesia and spinal anaesthesia is single-use plastics (20-25%), followed by electricity (15%) and pharmaceuticals (8%) | Lidocaine was found in high concentrations within aquatic invertebrates in the UK | A modified production method of sevoflurane with reduced reaction time and solvent requirement, which reduced the associated environmental impact | Only 25% of halothane, 3% of sevoflurane, 0.2% of isoflurane and 0.02% of desflurane is metabolised by the cytochrome p450 enzymes | 0.14–1.12ng/L ketamine was detected in drinking water samples from East Anglia, UK | Ozonisation shows a 16% removal of ketamine from fresh water and 5% removal of ketamine from wastewater | Lidocaine was found in 3 German wastewater treatment plant influents at a mean concentration of 134.67ng L^−1^, with a maximum removal efficacy of 50% | Lidocaine was found at a mean concentration of 176 ng L^−1^ across surface water samples in Germany | Validated the safety and feasibility of a membrane-based product as an alternative to chemical absorbers for use in scavenging systems with a lower environmental impact |
| **Life Cycle Domain(s) Studied** | Disposal of packaging | Excretion of medication and metabolites | Manufacturing Process | Intraoperative excretion or extraction | Disposal of drug | Excretion of medication and metabolites | Excretion of medication and metabolites | Excretion of medication and metabolites | Intraoperative excretion or extraction |
| **Studied Drug(s)** | Sevoflurane, Propofol | Ketamine, Lignocaine | Sevoflurane | Nitrous Oxide, Desflurane, Sevoflurane, Isoflurane, Halothane | Ketamine | Ketamine | Lignocaine | Lignocaine | Desflurane, Sevoflurane |
| **Type of study** | Environmental science paper | Environmental Science paper | Basic Science/chemistry | Review article | Environmental Science paper | Environmental Science paper | Environmental Science paper | Environmental Science paper | Poster |
| **Authors** | McGain et al., 2021 | Miller et al., 2019 | Moghimi et al., 2015 | Moppet et al., 2012 | Peng et al., 2019 | Rodayan et al., 2014 | Rua-Gomez et al., 2012a | Rua-Gomez et al., 2012b | Schmidt et al., 2019 |

| **Findings as reported in this review** | Sherman et al. (2012) modelled the production of desflurane, isoflurane, sevoflurane using data on catalysts and reagents derived from US patents^32^. Their results showed that greenhouse gas emissions of sevoflurane production is roughly half of that of desflurane, while the emissions for isoflurane production is roughly half again^32^. The manufacturing process of the medication was the largest contributor to the greenhouse gas emissions (not including release of waste gas) associated with the life cycle of desflurane and was the largest contributor for isoflurane and sevoflurane^32^. However, this was a very small contribution compared to the environmental impact of the release of waste anaesthetic gases in this study, although no exact values were provided^32^.  A second study included the carbon footprint of packaging of isoflurane, desflurane and sevoflurane in a life cycle analysis of greenhouse gas emissions, which showed packaging contributed a very small proportion of overall emissions^32^.  Two studies examined the distribution of anaesthetic medications^13,32^. Sherman et al. (2012) included road transport of anaesthetic medications in a modelling analysis, with anaesthetic gases being transported in lightweight polyvinyl chloride plastic containers and propofol transported in glass containers^32^. Although they provided no specific values on the environmental impact of supply chain, the analysis showed that transport makes up a very small proportion of the overall carbon footprint of anaesthetic medication use^32^.  One life cycle analysis examined wastage of propofol^32^. The authors assumed a 50% propofol wastage based on previous literature^32^. Although they state the total carbon dioxide equivalent of the life cycles of each medication, they do not state the specific values for each domain, including disposal of medication^32^. Nonetheless they do represent these values in a figure and disposal of medications is showed to represent a small proportion of the total carbon dioxide equivalent of propofol^32^. In addition, they state that unadministered propofol must be disposed of by incineration in accordance with pharmaceutical waste regulations and manufacturer recommendations, the environmental impact of which is unknown  In contrast, Sherman et al. (2012) concluded that packaging disposal constitutes a minimal proportion of the total greenhouse gas emissions of anaesthetic medications’ life cycles after accounting for the carbon footprint of waste management^32^.  Sherman et al. (2012) reported that waste anaesthetic gas release accounted for the majority of the total greenhouse gas emissions associated with the life cycle of desflurane, isoflurane and sevoflurane^32^ | Other studies found ketamine in 36 rivers in North China and in irrigation channels in Spain^33,34^ | Other studies found ketamine in 36 rivers in North China and in irrigation channels in Spain^33,34^ | Two studies in Beijing observed ketamine and it metabolites in sewage effluents and surface waters^7,35^ | Two studies in Beijing observed ketamine and it metabolites in sewage effluents and surface waters^7,35^ |
| --- | --- | --- | --- | --- | --- |
| **Relevant findings** | Desflurane has the largest life-cycle environmental impact (15 times that of isoflurane and 20 times that of sevoflurane). The main contributor to environmental impact of inhaled anaesthetic agents is released waste gases. The main contributor to the impact of propofol is electricity of syringe pump and packaging | Ketamine was found at a mean concentration of 21.33 ngL^−1^ across 23 sites in a wetland in Spain | Ketamine was found at a mean concentration of 0.49 ngL^−1^ across 36 rivers in China | Ketamine and norketamine were found in 13 sewage plant influents in Beijing with highest average concentration of 19.5±5.1 ngL^-1^ | High levels of ketamine were observed in urban areas of Beijing, with maximum mean concentration of 2.9 ngL^-1^ |
| **Life Cycle Domain(s) Studied** | Raw materials, Manufacturing process, Packaging of product, Distribution of drug, Disposal of drug after use, Intraoperative excretion or extraction | Excretion of medication and metabolites | Excretion of medication and metabolites | Excretion of medication and metabolites | Excretion of medication and metabolites |
| **Studied Drug(s)** | Nitrous Oxide, Desflurane, Sevoflurane, Isoflurane, Propofol | Ketamine | Ketamine | Ketamine | Ketamine |
| **Type of study** | Environmental Impact Assessment | Environmental Science paper | Environmental Science paper | Environmental Impact Assessment | Environmental Science paper |
| **Authors** | Sherman et al., 2012 | Vazquez-Roig et al., 2012 | Wang et al., 2016 | Zhang et al., 2016a | Zhang et al., 2016b |
